# Supplementary material for: Dynamic transcriptomic profiles of zebrafish gills in response to zinc supplementation
Source: BMC Genomics. 2010 Oct 11;11:553. doi: 10.1186/1471-2164-11-553 (PMC3091702; doi:10.1186/1471-2164-11-553)
Supplement: Additional file 2 — Interactive Direct Interaction Network representing the molecular interactions between zinc, copper, iron, calcium and proteins encoded by transcripts changed by zinc supplementation. Mini web-site containing index.html and hyperlinked pages in subdirectory describing a Direct Interaction Network automatically generated based on curated interactions contained within the proprietary PathwayArchitect database. Ovals represent proteins and the circles symbolize metal ions. Objects are coloured by their abundance in zebrafish at the time-point they were significantly different from the control is a scale from -4 fold (dark green) to +4 fold (dark red). Where significant differences were found at more than one time-point, the colour overlay shows expression at the first instance. Dark blue squares denote 'binding', and light blue squares 'expression'; green squares stand for 'regulation', green diamonds for 'metabolism', and green circles for 'promoter binding'. Arrow heads indicate directionality of the interaction where annotated. All nodes and edges can be further interrogated by selecting the relative area of the image. [file 1471-2164-11-553-S2.zip › PathwayArchitect Zn xs DIN/573450.html]

# REGULATION:

|  |  |
| --- | --- |
| Type | REGULATION |
| Effect | Positive |


---

|  |  |
| --- | --- |
| Score | 0 |


---

|  |  |
| --- | --- |
| Reference Count | 7 |


---

|  |  |
| --- | --- |
| Mechanism | Unknown |


---

|  |  |
| --- | --- |
| Reference:0 || Sentence | "This paper examines the development of iron (Fe) deficiency and its possible interactions with trace elements such as zinc (Zn) and copper (Cu) by investigating iron-deficient and control rats." |
| PMID | 9718478 |
| Year | 1998 |
| Species | Rat |
| Journal | Lab Anim |
| RefScore | 1 |
| Source | PArchNLP |
  |
|  |  |


---

|  |  |
| --- | --- |
 Reference:1 || Sentence | "CONCLUSIONS: Head neck cancer patients show the same characteristic trace element status as other solid tumors: decreased selenium, zinc and iron, increased copper." |
| PMID | 12915990 |
| Year | 2003 |
| Species | Human |
| Journal | Laryngorhinootologie |
| RefScore | 1 |
| Source | PArchNLP |
  ||  |  |


---

|  |  |
| --- | --- |
 Reference:2 || Sentence | "When the exposures were analyzed as continuous variables, the adjusted RRs (CI) were 1.66 (1.03-2.68) for 100 microg/dl iron increase, 1.17 (1.01-1.36) for 10% TS increase, 1.98 (1.12-3.50) for 100 microg/dl copper increase, and 0.57 (0.16-1.96) for 100 microg/dl zinc increase." |
| PMID | 15036223 |
| Year | 2004 |
| Species | Human |
| Journal | Ann Epidemiol |
| RefScore | 1 |
| Source | PArchNLP |
  ||  |  |


---

|  |  |
| --- | --- |
 Reference:3 || Sentence | "IL-1 reduced zinc and iron plasma levels and elevated copper plasma levels." |
| PMID | 8018447 |
| Year | 1993 |
| Species | Mouse |
| Journal | Biotherapy |
| RefScore | 1 |
| Source | PArchNLP |
  ||  |  |


---

|  |  |
| --- | --- |
 Reference:4 || Sentence | "After adjustment for age and time on dialysis, transferrin iron binding capacity (P = 0.013) and copper (P = 0.019) continued to be associated with CVD risk but ceruloplasmin (P = 0.065) and CRP (P = 0.634) were not." |
| PMID | 10945571 |
| Year | 2000 |
| Species | Human |
| Journal | Ann Clin Lab Sci |
| RefScore | 1 |
| Source | PArchNLP |
  ||  |  |


---

|  |  |
| --- | --- |
 Reference:5 || Sentence | "The results demonstrated that copper supplementation of human K562 cells was associated with higher cytosolic iron levels and ferritin levels." |
| PMID | 1508944 |
| Year | 1992 |
| Species | Human |
| Journal | Proc Soc Exp Biol Med |
| RefScore | 3 |
| Source | PArchNLP |
  ||  |  |


---

|  |  |
| --- | --- |
 Reference:6 || Sentence | However, she had normal liver iron content and increased liver copper content. |
| Year | 2006 |
| PMID | 16357618 |
| Species | Human |
| Journal | Eur J Gastroenterol Hepatol |
| RefScore | 2 |
| Source | PArchNLP |
  |


---

|  |  |
| --- | --- |
